# Nanoparticles: Alternatives Against Drug-Resistant Pathogenic Microbes

**DOI:** 10.3390/molecules21070836

**Published:** 2016-06-27

**Authors:** Gudepalya Renukaiah Rudramurthy, Mallappa Kumara Swamy, Uma Rani Sinniah, Ali Ghasemzadeh

**Affiliations:** 1 Department of Biotechnology, East-West College of Science, Bangalore-560091, Karnataka, India; murthygr123@gmail.com; 2 Department of Crop Science, Faculty of Agriculture, Universiti Putra Malaysia, Serdang, Selangor, Darul Ehsan 43400, Malaysia; upmali@yahoo.com

**Keywords:** nanoparticles, drug resistance, antimicrobial, mode of action, synthesis, silver, metal oxide, pathogens, antibiotics, medicine

## Abstract

Antimicrobial substances may be synthetic, semisynthetic, or of natural origin (i.e., from plants and animals). Antimicrobials are considered “miracle drugs” and can determine if an infected patient/animal recovers or dies. However, the misuse of antimicrobials has led to the development of multi-drug-resistant bacteria, which is one of the greatest challenges for healthcare practitioners and is a significant global threat. The major concern with the development of antimicrobial resistance is the spread of resistant organisms. The replacement of conventional antimicrobials by new technology to counteract antimicrobial resistance is ongoing. Nanotechnology-driven innovations provide hope for patients and practitioners in overcoming the problem of drug resistance. Nanomaterials have tremendous potential in both the medical and veterinary fields. Several nanostructures comprising metallic particles have been developed to counteract microbial pathogens. The effectiveness of nanoparticles (NPs) depends on the interaction between the microorganism and the NPs. The development of effective nanomaterials requires in-depth knowledge of the physicochemical properties of NPs and the biological aspects of microorganisms. However, the risks associated with using NPs in healthcare need to be addressed. The present review highlights the antimicrobial effects of various nanomaterials and their potential advantages, drawbacks, or side effects. In addition, this comprehensive information may be useful in the discovery of broad-spectrum antimicrobial drugs for use against multi-drug-resistant microbial pathogens in the near future.

## 1. Introduction

Antimicrobial agents kill or inhibit the growth of a wide range of microbes such as bacteria (antibacterials), fungi (antifungals), and viruses (antivirals). Antimicrobials may be synthetic, may be of plant or animal origin, or may be chemically modified natural compounds [1], and can have a significant impact on the outcome of an infected patient/animal. They are used in the treatment (chemotherapy) and prevention (prophylaxis) of infections. Many infectious diseases have been combatted since the discovery of antimicrobial drugs in the 1960s [2]. The history of antimicrobial agents dates back to 1928, when penicillin was discovered; however, it did not come into use until 1942. Penicillin was the first antibiotic to be used in medicine [3], and the management of life-threatening bacterial infections improved significantly after its discovery. Moreover, the discovery of penicillin, which is considered one of the most significant advances in medicine, started what is known as “the antibiotic revolution” [3,4,5]. Antimicrobials can be classified into one of several categories such as disinfectants, antiseptics, or antibiotics. Disinfectants and antiseptics are extensively and commonly used in hospitals and healthcare units, and are essential for the control and prevention of microbial infections. Various active chemical agents/biocides are used in the preparation of antiseptics and disinfectants. Antiseptics destroy (cidal) or inhibit (static) the growth of microorganisms in or on living tissue, whereas disinfectants, which are similar to antiseptics, are used on inanimate objects or surfaces.

The mode of action of antibiotics varies and includes the inhibition of cell wall synthesis, inhibition of protein synthesis, inhibition of DNA replication, and inhibition/alteration of intermediary metabolism [6]. The penetration of antimicrobials into the cell is necessary if the target for antimicrobial action is located inside the bacterial cell wall. Hence, antimicrobial agents must be capable of penetrating to the site of action. Penetration through the cell wall and/or membrane is usually achieved by passive or facilitated diffusion, or by an active transport mechanism. However, the presence of lipopolysaccharide-lipoprotein complexes in the cell wall of Gram-negative organisms prevents many antibiotics from reaching the sensitive intracellular targets. Some antibacterial agents use aqueous transmembrane channels (porins) in the outer membrane to gain entry into Gram-negative organisms. Peptidoglycan, which forms a rigid layer, is similar in both Gram-positive and Gram-negative bacteria with some differences. However, Gram-positive organisms possess a very thick peptidoglycan coat (cross-linked with interpeptide bridges) and Gram-negative organisms have a very thin peptidoglycan layer. Many antibiotics, such as penicillins, cephalosporins, fosfomycin, bacitracin, cycloserine, vancomycin, and teicoplanin, selectively inhibit the synthesis of the peptidoglycan layer at different stages [6]. Antimicrobials such as ionophores interfere with the transport of cations through the cell membrane. Gramicidin A, monensin, and valinomycin disturb cation transport, and antimicrobial peptides, such as defensins, cecropins, and magainins have ionophoric properties [7,8,9,10]. The intracellular targets for antimicrobials include protein synthesis and DNA replication. Antibiotics such as puromycin, chloramphenicol, tetracyclines, aminoglycosides, fusidic acid, lincosamides, macrolides, streptogramins, mupirocin, and oxazolidinones interfere with the process of protein synthesis. Modulation of DNA supercoiling by topoisomerases is an essential step in DNA replication. Several antibiotics target topoisomerases and inhibit DNA synthesis, thereby combatting many bacterial infections. The inhibitors of DNA synthesis include quinolones, novobiocin, rifampicin, diaminopyrimidines, sulfonamides, and 5-nitroimidazoles [11]. However, microorganisms revealed a remarkable capability to adapt, survive, and evolve by developing resistance to antimicrobial compounds. The improper use of antimicrobial agents has led to the development of new resistance mechanisms followed by the global spread of resistant organisms, which threatens the effective treatment of common infectious diseases. One of the greatest challenges for practitioners is the emergence of multi-resistant bacteria, such as methicillin-resistant *Staphylococcus aureus* (MRSA) and vancomycin-resistant enterococci; this constitutes a global health threat. The health risk for patients infected with resistant bacteria is higher than those infected with non-resistant bacteria, and it results in prolonged illness and higher expenditure. Furthermore, the major concern/risk with the development of antimicrobial resistance is the spread of resistant organisms. MRSA strains, in hospitals or from clinical sources, have recently been detected in workers involved in animal production as well as within community settings [12].

The development of antimicrobial resistance may be caused by: (a) alteration or inactivation of the drug; (b) reduced binding capacity of the drug due to alteration in the binding sites; (c) reduction in the antimicrobial effect due to modification of the metabolic pathways; or (d) decreased permeability and/or increased active flux leading to reduced intracellular accumulation of antimicrobial agents [13]. However, antimicrobial resistance may be intrinsic or acquired; it can develop through the mutation of existing genes [14,15], or through the transfer of genes from other species or strains [16,17]. Antimicrobial resistance can be detected by growth inhibition assays in broth or agar disc diffusion [13]. Culture-based assays may be fast or slow depending on the doubling time of microbes. However, culture-based assays are not suitable for the vast majority of microbes that cannot grow outside the host, such as *Mycobacterium leprae*, *Treponema pallidum*, *Corynebacterium diphtheria*, *Bartonella* *henselae*, *Tropheryma whippelii*, and noroviruses. Molecular detection techniques such as diagnostic polymerase chain reaction (PCR) assays [18,19], quantitative PCR [20], and DNA microarrays [21,22] significantly improved disease diagnosis and the identification of resistance genes. Moreover, antibiotic resistance genes from unculturable microbes can be identified through metagenomics. The authors of several earlier studies report the identification of resistance genes such as those that encode β-lactams and bleomycin [23,24,25]. Fighting antibiotic resistance is a major priority in human and animal health. Several strategies are available to overcome antibiotic resistance, including a reduction in the extensive use of antimicrobials, collection and analysis of data, avoiding the inappropriate use of antimicrobials in farm animals, development of novel drugs, and nanotechnology [26,27]. Advances in nanotechnology have led to the synthesis of nano-sized organic and inorganic molecules with potential applications in industry, food packaging, textiles, medicine, and therapeutics. The development of novel nanoscale antimicrobial agents/nanocomposites can be used as an alternative strategy to overcome antimicrobial resistance [27].

The advent of nanotechnology, the biggest engineering innovation of recent times, has modernized medicine. The demand for nanotechnology-derived products is constantly increasing. Nanotechnology, which is the innovative technology in the present scenario, can have a profound influence on improving human health. Enhanced durability, performance, strength, flexibility, and the inimitable physicochemical properties of nanomaterials have been explored in the health industry. Nanomaterials can be used in treatment modalities including targeted drug delivery, prognostic visual monitoring of therapy, and even the detection of tumors [28,29]. However, continuous exposure of humans to nanoparticles (NPs) in the work place can cause unpredictable human health risks. Moreover, indirect exposure to NPs occurs when they are inhaled as air pollutants. Inhaled NPs sometimes evade the immune system and are distributed throughout the body, causing systemic health problems. The pollution of air by NPs is even detrimental to other biological species in the environment and disturbs the ecosystem [30]. Serious health problems due to ambient or occupational exposure may arise if these issues are not addressed in a cohesive and concerted manner by industries, governments, and scientists. In light of this, the present review was conducted to present complete information on the antimicrobial activity of different types of nanomaterials. In addition, we emphasized the application of NPs/nanocomposites in combating antimicrobial drug resistance. This review is a compiled survey of data retrieved from many search engines including ScienceDirect, Google Scholar, PubMed, Scopus, and SciFinder.

## 2. Nanoparticles/Nanocomposites

**Figure 1:**
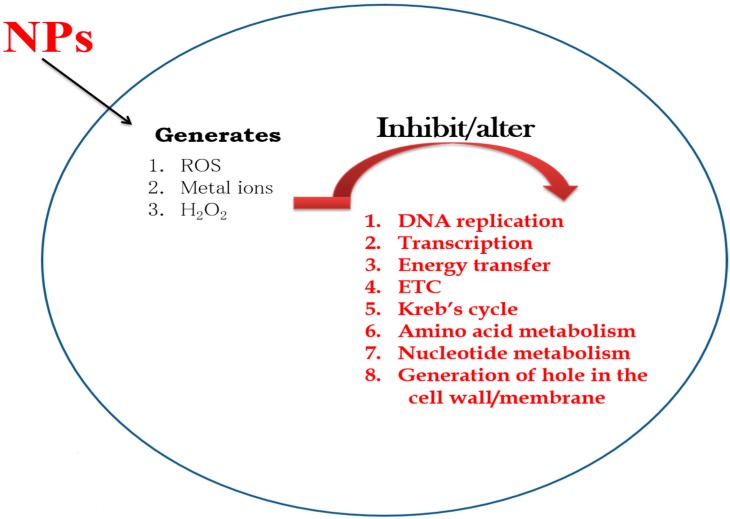
Mechanism of action of various nanoparticles (NPs) on microbial cells.

**Figure 2:**
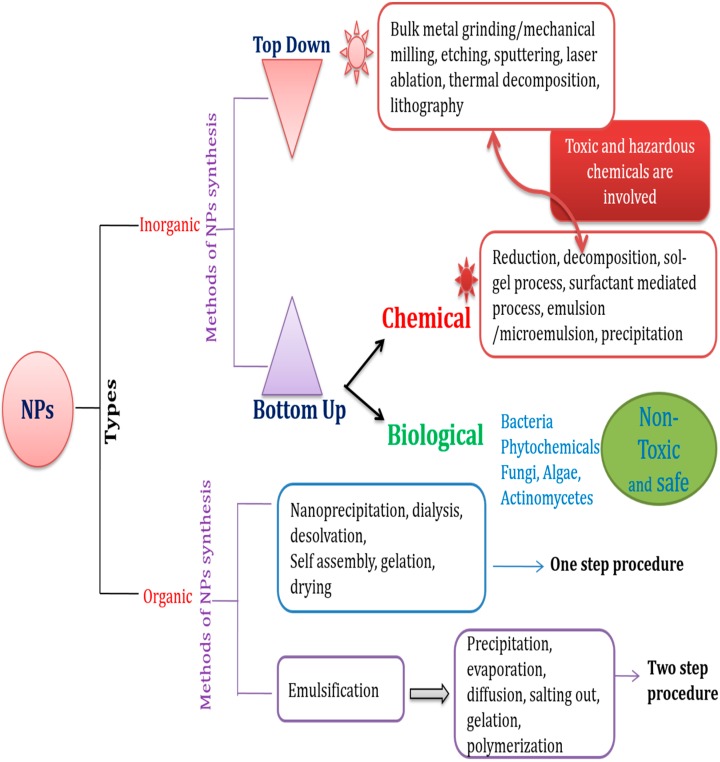
Schematic representation of the synthesis of nanoparticles (NPs) by various methods.

Antimicrobial drug resistance has prompted the development of several alternative strategies. Among these strategies, nanoscale materials/nanocomposites have emerged as significant and novel antimicrobial agents. Nanomaterials, typically 0.2–100 nm in size, have a high surface-to-volume ratio [31]; this increases their interaction with microorganisms, which in turn improves their antimicrobial activity. Transmission electron microscopy (TEM), low-resolution TEM (LRTEM), and high-resolution TEM (HRTEM) have helped in the characterization of NPs and revolutionized their use in various fields. The chemical, electrical, mechanical, optical, magnetic, and electro-potential properties of NPs differ from those of their bulk materials. This may be attributed to their high surface-to-volume ratio [32,33]. The physicochemical and biological properties of NPs can be manipulated according to the desired application [31,34]. NPs may be organic or inorganic; however, inorganic NPs are used more often owing to their ability to withstand adverse reaction conditions [31]. NPs have been used in optical, chemical, and biological fields. Their potential applications include many specific areas such as superconductors, optical devices, catalysts, fuel cells, gene and drug delivery, cell and tissue imaging, and biosensors [35,36,37,38,39]. Moreover, NPs have antimicrobial properties and have potential for use in diagnostic immunoassays [40,41,42]. Several types of NPs, including various metal and metal oxides, have been developed and evaluated by different research groups; examples include silver (Ag), gold (Au), Ag oxide (Ag_2_O), zinc oxide (ZnO), titanium dioxide (TiO_2_), calcium oxide (CaO), copper oxide (CuO), magnesium oxide (MgO), and silicon dioxide (SiO_2_) [39]. NPs act on microbes by several different methods and the mode of action of these NPs varies with each different type (Table 1 and Figure 1). Several bacterial strains are capable of adhering to any natural or artificial surface, and can even form biofilms on these surfaces. Many different factors are responsible for the adhesion and formation of biofilms by bacteria; these include the production of slime-like substances, electrostatic interactions, dipole-dipole and H-bond interactions, hydrophobic interactions, and van der Waals interactions. Therefore, nanomaterials that are used as antimicrobial agents must reduce microbial adhesion and biofilm formation. Hence, screening NPs for their anti-adhesion capability would increase their potential as antimicrobial agents. A schematic representation describing the various methods for the synthesis of different NPs is shown in Figure 2.

### 2.1. Inorganic NPs with Antibacterial and Antifungal Activities

Several inorganic metals and their oxides, including Ag, TiO_2_, CuO, iron oxide (Fe_3_O_4_), and ZnO, have been studied for their antimicrobial activities.

#### 2.1.1. Silver NPs (AgNPs)

AgNPs are synthesized by physical, chemical, and biological methods. The physical method, which is also known as the “top-down” method, involves grinding the bulk metal, whereas the chemical method, widely called the “bottom-up” method, involves reduction, electrochemical processes, and decomposition by ultrasonic waves [43,44,45]. However, the synthesis of AgNPs by physical and chemical processes involves the use of toxic and hazardous chemicals, and the process is extremely expensive. The biological method, which is a “bottom-up” approach, exploits bacteria, fungi, and plant extracts to synthesize NPs. Recently, biologically synthesized NPs have received a great deal of attention, mainly in the field of biomedicine [46]. The biological method involves oxidation or reduction reactions by enzymes produced by microorganisms, or by phytochemicals. Several bacteria, fungi, and plants including *Pseudomonas stutzeri*, *Bacillus megaterium*, *Escherichia coli* [47,48,49], *Aspergillus fumigatus*, *Fusarium solani* [50,51], *Aloe vera*, *Piper betle* leaf, *Leptadenia reticulata*, and *Momordica cymbalaria*, have been explored for use in the synthesis of AgNPs [27,44,52,53,54,55,56]. The size of the AgNPs synthesized by biological methods varies between 1 and 600 nm [27,43,54].

The antimicrobial activities of Ag, Ag ions (Ag^+^), and Ag compounds have been known for many centuries. Ag has broad-spectrum antimicrobial activity against bacteria, fungi, and viruses, which is termed “oligodynamic activity”. Ag and its compounds undergo ionization in water and/or in body fluids, and the bioactive Ag^+^ interact with proteins and amino acids. Microorganisms are highly susceptible to the toxic effect of Ag^+^ and Ag compounds [57]. The mechanism of antimicrobial activity of Ag^+^ involves interference with the electron transport chain and the transfer of energy through the membrane, because Ag has an affinity for the sulfhydryl (thiol) groups in cell wall enzymes [57,58]. Ag^+^ also inhibit DNA replication and the respiratory chain in bacteria and fungi. However, the antimicrobial activity of Ag and its compounds is directly proportional to the number of biologically active Ag^+^ released, and its availability for interaction with the bacterial cell wall [57]. AgNPs are a good source of antimicrobial agents and possess antioxidant, anti-inflammatory, anticancer, and antiangiogenic activities [27,31,44,55]. The bactericidal activity of AgNPs against several pathogenic bacteria has been investigated by many research groups [27,31,43,44,54,59,60,61,62,63]. At present, AgNPs are widely considered an alternative antibacterial agent to Ag^+^. This is because the effects of Ag^+^ have a limited duration. AgNPs exhibit superior antimicrobial properties mediated by the synthesis of reactive oxygen species (ROS) including hydrogen peroxide [43,45,55]. Furthermore, the larger surface-to-volume ratio of AgNPs allows increased interactions with the cell membrane and easy penetration into the cell, leading to complete destruction of microbial cells compared with Ag^+^ [57,64].

Silver ions have a high affinity for sulfur and phosphate groups, which might explain their antimicrobial activity. Ag^+^ released from NPs react with sulfur-containing proteins, mainly on the cell surface, and phosphorous-containing nucleic acids. They are known to produce ROS inside the cell, eventually leading to cell death [31,57,59,92]. Nucleic acid damage and the alteration of the bacterial cell wall brought about by the attachment of AgNPs are considered the major reasons for bacterial cell death [45,92]. The size and shape of NPs play a significant role in their antimicrobial activity. AgNPs with a diameter of ≤10 nm form pores in the cell wall leading to the death of an organism [31,43,44,54,57]. The minimum inhibitory concentration (MIC) varies with the size of the NPs. The MIC of NPs smaller than 25 nm is 6.75–54 μg/mL, whereas 25-nm particles have a lower MIC of 1.69–13.5 μg/mL against methicillin-resistant *Staphylococcus aureus* and *Staphylococcus epidermidis*, and vancomycin-resistant *Enterococcus faecium* and *Klebsiella pneumonia* [31,57]. A Gram-positive bacterium, *S. aureus*, was effectively inhibited by AgNPs at higher concentrations (100 μg/mL) [60]. In addition, Rupareli et al. [82] observed strain-specific variations in the MIC/minimum bactericidal concentration (MBC) of *E. coli* when treated with AgNPs. The MIC values ranged from 40 to 180 μg/mL for different strains of *E. coli* (MTCC 443, MTCC 739, MTCC 1302, and MTCC 1687).

According to Lara et al. [93], AgNPs exhibited high bactericidal activity against multidrug-resistant *Pseudomonas aeruginosa*, ampicillin-resistant *E. coli* O157:H7, and erythromycin-resistant *Streptococcus pyogenes*. The MIC was 83.3 mM for *P. aeruginosa* and *E. coli* O157:H7, whereas it was 83.3 mM for *S. pyogenes*. They also suggested the possible use of AgNPs as a potential antimicrobial agent in medical devices, pharmaceutical products, and in the nosocomial environment. Likewise, Morones et al. [65] reported the antibacterial activity of AgNPs against Gram-negative bacteria, such as *E. coli*, *P. aeruginosa*, *V. cholera*, and *S. typhus*. Many other pathogenic bacteria are susceptible to AgNPs; they include *Acinetobacter baumannii*, *Enterococcus faecalis*, *Klebsiella pneumoniae*, *Listeria monocytogenes*, *Micrococcus luteus*, *Proteus mirabilis*, *Salmonella typhi*, *Enterobacter aerogenes*, *Bacillus subtilis*, *Brucella abortus*, *Moraxella catarrhalis*, *Proteus mirabilis*, *Streptococcus viridans*, *Streptococcus pneumonia*, *Streptococcus mutans*, *Serratia proteamaculans*, and *Shigella flexneri* [27,43,45,53,54,57,59,94]. More recently, it has been reported that AgNPs kill *S. mutans* isolated from clinical samples, suggesting their use for the treatment of dental caries [94]. Furthermore, fungal infections markedly contribute to increasing the mortality and morbidity of immunocompromised patients. Several studies have shown that AgNPs act as a potential antifungal agent. The antifungal activity of AgNPs is influenced by their size and zeta potential. Moreover, the mechanism of inhibition against fungi varies with particle size. AgNPs were found to be effective against various fungal pathogens, including *Candida albicans*, *Candida tropicalis*, *Trichophyton rubrum*, *Penicillium brevicompactum*, *Cladosporium cladosporioides*, *Aspergillus fumigatus*, *Chaetomium globosum*, *Mortierella alpina*, and *Stachybotrys chartarum* [95,96,97,98].

#### 2.1.2. Magnesium Oxide (MgO) NPs

Inorganic metal oxides such as MgO, ZnO, and CaO are stable under harsh processing conditions and are generally considered safe for humans [99]. Moreover, MgO does not require photoactivation for its antimicrobial activity [100,101,102,103]. Various strategies have been developed for the synthesis of MgONPs that are similar to those used to develop AgNPs. The regulation of processing conditions allows the synthesis of MgONPs of various sizes and with different morphologies [104,105]. Several mechanisms have been proposed to explain the antibacterial activity of MgONPs, which include the formation of ROS, lipid peroxidation, electrostatic interactions, and alkaline effects. The strong electrostatic interaction between the bacterial cell surface and the MgONPs leads to the death of the bacteria [101]. The surface of MgONPs has a typically high pH due to the formation of a thin layer of water. When bacteria contact MgONPs, the high pH damages the bacterial cell membrane (the alkaline effect), ultimately leading to death. However, the antibacterial efficacy of MgONPs is size-dependent owing to changes in the surface energy. Particles smaller than 15 nm exhibit higher bactericidal activity than large, aggregated MgONPs [106,107]. MgONPs exhibit antibacterial activity against Gram-positive and Gram-negative bacteria such as *S. aureus* (MIC; 1000 μg/mL), *E. coli* (MIC; 500 μg/mL), and *P. aeruginosa* (MIC; 1000 μg/mL) [66]. MgONPs synthesized by the aerogel procedure exhibit biocidal activity against vegetative forms of Gram-positive and Gram-negative bacteria, and against spores, and can also be used as a potent disinfectant [67,108]. Moreover, MgONPs exhibit antimicrobial activity against *E. coli*, *Bacillus megaterium*, and *B. subtilis* [109].

#### 2.1.3. Titanium Dioxide (TiO_2_) NPs

TiO_2_ is a non-toxic and chemically stable molecule with optical properties [109]. TiO_2_ NPs (TiO_2_NPs) can be used in pharmaceuticals, cosmetics, whiteners, food colorants, toothpaste, and to protect the skin against UV rays [110]. Moreover, they have significant antibacterial activity against certain microbes [111]. Several methods are available for the synthesis of TiO_2_ NPs including sol-gel and electrochemical techniques. Like other metals or metal oxides, TiO_2_ acts on bacteria through the generation of ROS. The oxidative stress on the crystal surfaces of anatase TiO_2_ generates ROS. The surface of anatase TiO_2_ reacts with water by photocatalysis and releases the hydroxyl radicals, which subsequently form superoxide radicals [112]. The ROS synergistically act on phospholipids (polyunsaturated) on the surface of bacteria [113], causing site-specific DNA damage [68,69]. The photocatalytic activity of TiO_2_ can be exploited in the preparation of biofilms, which have many applications such as the disinfection of contaminated surfaces in food processing industries. The antimicrobial activity of photocatalytic TiO_2_ against *E. coli*, *S. aureus*, and fungi was reported [31]. Researchers reported the light-induced biocidal activity of engineered TiO_2_ NPs against *E. coli* [114] and *Aspergillus niger* [115].

#### 2.1.4. Zinc Oxide (ZnO) NPs

The U.S. Food and Drug Administration listed ZnO as “generally recognized as safe” (GRAS) [116]. The application of ZnO NPs (ZnONPs) depends on their shape, size, surface state, crystal structure, and dispensability. Several different techniques have been developed for the synthesis of ZnONPs, which include mechanochemical, precipitation, emulsion, and microemulsion processes. ZnONPs occur as different structures such as nanorods, needles, helixes, springs, rings, nanoplates/nanosheets, nanopellets, flowers, dandelions, and snowflakes. The mechanism of ZnONP antimicrobial activity involves the generation of hydrogen peroxide and the release of Zn^2+^ ions. ZnO generates highly reactive oxygen species such as OH^−^ and hydrogen peroxide (H_2_O_2_). Hydrogen peroxide generated on the surface of ZnO can penetrate the bacterial cells and effectively inhibit cell growth [70,71]. However, OH^−^ and superoxides are likely to remain on the surface of the cell because they cannot penetrate the cell membrane owing to their negative charge. The generation of hydrogen peroxide increases with the increasing surface area of the ZnONPs. However, Zn^2+^ ions released from the NPs damage the cell membrane and interact with intracellular components [72]. A wide range of Gram-positive and Gram-negative bacteria, including major foodborne pathogens, is susceptible to ZnONPs. Previous studies have shown that ZnONPs exhibit antibacterial activity against *E. coli*, *Listeria monocytogenes*, *Salmonella*, and *Staphylococcus aureus* [73,74]. In a study by Vidic et al. [117], ZnO nanostructures (100 nm) showed effective antibacterial activity against both Gram-positive (*B. subtilis*) and Gram-negative (*E. coli*) bacteria. However, at a lower concentration of 1 mg/mL, 100% inhibition was observed. Likewise, Reddy et al. [118] reported the inhibition of *E. coli* (~13 nm) by ZnONPs at ≥3.4 mM concentration, while *S. aureus* was completely inhibited at ≥1 mM concentration. Polyethylene glycol (PEG)-capped ZnONPs at above 5 mM concentration showed antibacterial activity against *E. coli* [119]. Later, Li et al. [120] suggested that the toxicity of ZnONPs arises mostly from the labile zinc complexes and free zinc ions. Pati et al. [121] reported the potential application of ZnONPs as antimicrobials against *S. aureus.* The mechanism of the ZnONP antimicrobial activity is related to the disruption of the bacterial cell membrane integrity, the diminishing cell surface hydrophobicity, and the downregulation of the transcription of oxidative stress resistance genes in bacteria. ZnONPs also induce ROS production to augment intracellular bacterial death. In another study, the MIC of ZnONPs for *Campylobacter jejuni* was reported at 0.05 to 0.025 mg/mL concentration [116].

#### 2.1.5. Iron Oxide (Fe_3_O_4_) NPs

The intrinsic properties of iron-containing NPs increase their scientific, technological, and industrial value. Fe_3_O_4_ NPs are used in biosensors, food preservation agents, antimicrobial agents, magnetic refrigeration and storage media, ferrofluids, anti-cancer agents, magnetic resonance imaging (MRI), targeted drug delivery, and cell sorting. The biological compatibility and magnetic properties of Fe_3_O_4_ NPs make them attractive for applications in biomedical research [122,123]. Several different approaches are available for the synthesis and characterization of Fe_3_O_4_ NPs, including techniques such as sol-gel and forced hydrolysis, co-precipitation, hydrothermal processing, surfactant-mediated synthesis, laser pyrolysis, electrochemical processing, and microemulsion processing. Techniques such as absorption spectrophotometry, X-ray diffraction, and scanning electron microscopy (SEM) are available for the characterization of the synthesized NPs. The mode of antimicrobial action of Fe_3_O_4_ NPs might be through ROS, oxidative stress, superoxide radicals (O^2^^−^), singlet oxygen (^1^O_2_), hydroxyl radicals (OH^−^), or hydrogen peroxide (H_2_O_2_) [83]. The antimicrobial activity of Fe_3_O_4_ NPs against various bacteria including *S. aureus*, *S. epidermidis*, *E. coli*, *Xanthomonas*, and *P. vulgaris* has been established by several groups [124,125,126,127]. Chen et al. [123] demonstrated that immunoglobulin G-bound Fe_3_O_4_/titania core/magnetic shell NPs effectively inhibit the growth of various pathogenic multi-antibiotic-resistant bacteria such as *Staphylococcus saprophyticus*, *S. pyogenes*, and MRSA. Furthermore, Arokiyaraj et al. [128] evaluated the antimicrobial efficiency of Fe_3_O_4_ NPs, *Argemone Mexicana* L. plant leaf extract and Fe_3_O_4_ NPs treated with plant leaf extract against bacterial pathogens. Interestingly, they observed a considerable inhibition of *E. coli* MTCC 443 and *P. mirabilis* MTCC 425 strains by Fe_3_O_4_ NPs treated plant extract. According to Anghel et al. [129], Fe_3_O_4_ NP-coated textile dressings inhibit biofilm formation by *C. albicans* more than uncoated textile dressings. Moreover, Fe_3_O_4_ NPs coated with *Rosmarinus officinalis* essential oil had potent inhibitory activity against biofilm-forming *C. albicans* and *C. tropicalis* [130].

#### 2.1.6. Gold (Au) NPs

Historically, colloidal Au was thought to have healing properties when consumed orally, and it is the earliest recognized form of AuNPs. The unique optical properties of AuNPs make them attractive potential tools in biomedicine. AuNPs are inert, biologically compatible, and have a high surface-to-volume ratio. The properties of AuNPs are very different from Au in its bulk form [131]. AuNPs can be prepared in a variety of different shapes or geometries such as nanorods, nanocages, nanocubes, and nanotriangles, which affect their optical features [132,133,134,135]. AuNPs in the size range 0.8–250 nm are regarded as the most popular NPs. However, nanoshells, which range in size from 80 to 150 nm, and smaller nanoshells (20–60 nm) containing a core of Fe_3_O_4_ nanocrystals, have also been extensively explored in biomedicine [136,137]. AuNPs can be synthesized by both chemical and biological methods. Chemical methods include the reduction of tetrachloroauric acid to produce colloidal AuNPs (size range 10–60 nm), the Brust–Schiffrin method for thiolated AuNP synthesis (size range 1–6 nm), Au nanoshell synthesis, and the seed-mediated method for the synthesis of nanorods (size range 1–2 nm) [133,134]. Though several chemical methods are available for synthesis, they are expensive and involve toxic chemicals. To overcome the limitations of chemical methods, several groups have developed economically feasible and eco-friendly biological methods for the synthesis of AuNPs [138]. Several researchers have used a biological method to synthesize AuNPs of various sizes and shapes, and tested their antimicrobial activity. For instance, they synthesized AuNPs using the bacteria *Rhodopseudomonas capsulata* or the fungus *C. albicans*, or used plant extracts as the reducing and stabilizing agents [138,139,140,141,142,143]. AuNPs are biologically inert, but they can be modified so that they have various functional groups, such as chemical or photothermal functionalities. Au nanorods are reported to have anti-cancerous and antimicrobial activity following photo-thermal heating [31]. AuNPs in combination with photosensitizers such as toluidine blue O are reported to exhibit antimicrobial activity against MRSA [75,76,77]. Biomolecules such as carbohydrates, antibodies, proteins, and oligonucleotides can be attached to AuNPs as functional moieties [144]. The addition of functional moieties increases the antimicrobial efficacy of NPs and several such modifications have been developed. AuNPs conjugated with specific antibodies have been reported to kill *S. aureus* (photothermally, using lasers) [145]. Antibiotics such as vancomycin, used to kill vancomycin-resistant enterococci [146,147], and aminoglycosidic antibiotics that act on both Gram-positive and Gram-negative bacteria [78,148,149], have been added to AuNPs. AuNPs act on bacteria through the generation of holes in the cell wall, which eventually lead to cell death due to the leakage of cell contents. Moreover, AuNPs can bind to DNA and inhibit the transcription process by preventing the uncoiling of DNA during transcription [78]. Khan et al. [150] reported the possible use of AuNPs (21 ± 2.5 nm and 0.2 mg/mL) conjugated with methylene blue (20 μg/mL) for preventing the formation of biofilm by the common nosocomial refractory fungus *C. albicans*. Several multidrug-resistant uropathogens, namely *E. coli*, *E. cloacae* complex, *P. aeruginosa*, *S. aureus*, and *S. aureus-*MRSA, were completely inhibited by AuNPs at nanomolar (8–32 nM) concentrations [151]. Mixed ligand-coated AuNPs have shown 99.9% growth inhibition against methicillin-susceptible *S. aureus* at a concentration of 10 μM [152]. Likewise, AgNPs synthesized biologically from the fungus *Trichoderma viride* showed MIC values of 40, 1.5, and 8 µg/mL against *E. coli* ATCC 8739, vancomycin-sensitive *S. aureus* ATCC 6538, and vancomycin-resistant *S. aureus*, respectively [153]. Au nanospheres conjugated with gentamycin showed enhanced antibacterial effect (0.0937 mg/mL MIC) against *S. aureus* compared with free gentamicin (0.18 mg/mL MIC) [154]. In a recent study, stable biofabricated AuNPs conjugated with gentamycin, ciprofloxacin, rifampicin, and vancomycin effectively inhibited *S. epidermidis* and *Staphylococcus haemolyticus* compared with antibiotics alone [155]. Dasari et al. [156] reported the effectiveness of AuNPs and Au ion complexes against three multidrug-resistant bacteria, namely *E. coli*, *Salmonella typhimurium* DT104, and *S. aureus*.

#### 2.1.7. Copper Oxide (CuO) NPs

Copper and its compounds have wide potential applications in several fields owing to their wide range of physical properties, namely, superconductivity, high thermal conductivity, spin dynamics, and electron correlation effects. CuO is a semiconducting compound. Its monoclinic structure has photoconductive and photocatalytic or photovoltaic properties [31,157]. Several methods have been reported for the synthesis of CuONPs, which include laser irradiation, γ-radiolysis, thiol-induced reduction, reverse micelles, and green synthesis [79,158,159]. Several studies have reported that copper NPs exhibit antibacterial activity against Gram-positive bacteria, including *B. subtilis* and *S. aureus*, and Gram-negative bacteria, including *E. coli* [80,82]. Copper NPs synthesized by a biological process were reported to have antibacterial activity against the human pathogens *E. coli* and *S. aureus* [80]. The mechanism of the antibacterial activity of CuONPs involves the adhesion of NPs to bacterial cell walls, owing to opposite electric charges, which results in reduction at the cell wall of the bacteria. In addition, Cu^2+^ ions generate ROS resulting in oxidative stress-induced DNA and membrane damage to the bacteria [81,160,161]. Moreover, CuONPs have a strong affinity for the amines and carboxyl groups present on the cell surface of *B. subtilis*, which might explain their high antimicrobial activity against such organisms. More recently, many pathogens such as *Klebsiella aerogenes*, *Pseudomonas desmolyticum*, *E. coli* (Gram-negative) and *S. aureus* (Gram-positive) have been effectively inhibited using CuONPs synthesized from *Gloriosa superba* L. plant extracts [162]. Likewise, Khashan et al. [163] reported the antibacterial effect of CuONPs against *E. coli*, *P. aeruginosa*, *P. vulgaris*, and *S. aureus.* CuONPs, when combined with fluconazole, have shown improved antifungal activity against *C. albicans* [161].

#### 2.1.8. Aluminum (Al) NPs

Aluminum oxide (Al_2_O_3_) or alumina, generally referred to as corundum (the crystalline form of alumina), is a white oxide with several phases: alpha, gamma, delta, and theta. Alpha-phase AlNPs are thermodynamically stable over a wide temperature range. In AlNPs, oxygen atoms adopt hexagonal close packing, and in the octahedral sites Al^3+^ ions fill two-thirds of the lattice to form a corundum-like structure [164]. Several different techniques are available for the synthesis of alumina NPs such as sol-gel pyrolysis, hydrothermal processing, sputtering, and laser ablation. The laser ablation technique is a rapid and high-purity process; hence, it is most widely used in the preparation of Al_2_O_3_ NPs [165]. Al_2_O_3_ and AlNPs have a wide range of applications in industry and medicine. Few studies have addressed the antimicrobial properties of AlNPs. According to Jing et al. [166], AlNPs had higher toxicity against *B. subtilis*, *E. coli*, and *Pseudomonas fluorescens* than their bulk materials. Similarly, alumina NPs have shown higher sensitivity and mutagenicity against *P. fluorescens* than the bulk materials [167]. AlNPs disrupt bacterial cell walls leading to cell death through ROS [168]. Sadiq et al. [84] reported that alumina NPs exhibit a growth-inhibitory effect on *E. coli* over a wide concentration range (10–1000 μg/mL).

#### 2.1.9. Bismuth (Bi) NPs

Bismuth, a diamagnetic, crystalline, and brittle metal, is typically found as bismuth sulfide (bismuthinite), bismuth oxide (bismite), and bismuth carbonate (bismuthite) [169]. Bismuth and its compounds exhibit antimicrobial activity against various bacteria. Bismuth compounds are commonly employed in the treatment of gastrointestinal disorders. The antimicrobial activity of elemental bismuth is observed at relatively high concentrations owing to its limited water solubility. However, effective antimicrobial activity at lower concentrations can be achieved by increasing the solubility of bismuth with chelating agents such as dimercaptopropanol. Bismuth-dimercaptopropanol has high solubility and decreases antimicrobial activity for short periods. Hence, the slow dissolution of bismuth-dimercaptopropanol would enable antimicrobial activity for an extended period [170]. Bismuth NPs (BiNPs) are synthesized from commercial bismuth salts using surface modifiers and a suitable reducing agent. BiNPs exhibit antifungal, antibacterial, and antiviral activity. Earlier studies by Hernandez et al. [171,172] reported that BiNPs exhibit antibacterial (<1 mM) and antifungal (2 mM) activity at lower concentrations. BiNPs have potential in the treatment of drug-resistant bacteria [85], and inhibit the growth of *Helicobacter pylori* through alteration of the Krebs cycle and amino acid and nucleotide metabolism [86]. BiNPs are promising candidates for fighting many infectious diseases. However, their safe use in humans requires evaluation.

#### 2.1.10. Carbon-Based NPs

Carbon-based nanomaterials play a significant role in biomedical applications such as tissue regeneration, advanced imaging, and drug or gene delivery. Several different types of carbon-based NPs have been developed including fullerenes, carbon nanotubes, and graphene oxide. The physical orientation of the carbon-based NPs and their particular use, depends on the conditions used to synthesize them [173]. The size and surface area of carbon-based NPs are relevant to their antibacterial activity; increased antimicrobial activity is associated with increased nanoparticle surface area and decreased size. Moreover, the antimicrobial activity also depends on the composition, intrinsic properties, and surface modification of the NPs [87,174]. The mode of action of carbon-based NPs includes severe damage to the bacterial membrane through oxidative stress [175,176] and/or physical interaction [88,176], the inhibition of energy metabolism [89,177], or impairment of the respiratory chain [178]. Direct contact between carbon nanomaterials and bacterial cells due to aggregation can also lead to cell death [87,88]. Carbon nanotubes are active against *E. coli* [87]. Single-walled carbon nanotubes (SWNTs) and multi-walled carbon nanotubes (MWNTs) have shown antimicrobial activity against both Gram-positive and Gram-negative bacteria owing to cell wall damage and the subsequent release of cell DNA [179]. Moreover, SWNTs exhibit antimicrobial activity against *Salmonella enterica*, *E. coli*, and *E. faecium* [90]. Fullerenes, which are ball-shaped carbon NPs, have antimicrobial activity against *E. coli*, *Salmonella*, *Streptococcus* spp., and *Shewanella oneidensis* [180,181]. Graphene, a two-dimensional crystal, and graphene oxide were shown to have an inhibitory effect on *E. coli* and *S. aureus* [182]. Carbon NPs complexed with Ag were found to have effective antimicrobial activity against MRSA, multidrug-resistant *Acinetobacter baumannii*, *Burkholderia cepacia*, *Yersinia pestis*, and *Klebsiella pneumoniae* [91]. The size of carbon nanotubes plays a significant role in governing their antibacterial properties [87,183,184]. Well-characterized SWNTs possess higher antibacterial activity than MWNTs. High levels of stress-related gene expression products were observed in *E. coli* when treated with MWNTs or SWNTs by Kang et al. [184]. Later, Vecitis et al. [185] proposed a possible antimicrobial mechanism of SWNTs involving the initial contact of SWNTs with bacteria (*E. coli*) followed by the perturbation of the cell membrane and electronic structure-dependent bacterial oxidation.

### 2.2. Organic NPs

Organic antimicrobials are considered less stable in nature compared with inorganic antimicrobials. Organic materials are highly susceptible to harsh process conditions, mainly at higher temperatures; this may lead to difficulties in designing and synthesizing organic NPs. However, several polymeric organic NPs that exhibit antimicrobial activity have been developed, including quaternary ammonium compounds, quaternary phosphoniums, and alkyl pyridiniums [186]. Polymeric NPs work either by releasing potent antimicrobial agents or by contact killing. They are chemically stable, nonvolatile, and can bind to the surface of cells. However, they cannot easily permeate through biological membranes [187]. Several organometallic polymers containing metals in the backbone chain or in the pendant groups have been synthesized by varying the size of the alkyl groups (methyl, ethyl, butyl, and octyl) [186]. These organometallic polymers exhibit effective inhibition against both DNA and RNA viruses. Several peptides have been synthesized using hydrophilic (lysine) and hydrophobic (alanine, phenylalanine, and leucine) amino acids. Peptides synthesized using lysine and phenylalanine exhibit broad and effective antimicrobial activity against *E. coli*, *P. aeruginosa*, *Serratia marcescens*, and *C. albicans* [186,188].

#### 2.2.1. Quaternary Ammonium Compounds

Quaternary ammonium compounds (QAC) include well-known disinfectants such as cetrimonium chloride, benzalkonium chloride, and stearalkonium chloride. The *N*-alkyl chain length determines the antimicrobial activity of QACs. Alkyl chains with 12–14 carbons are effective against Gram-positive bacteria, whereas alkyl chains with 14–16 carbons have significant antibacterial activity against Gram-negative bacteria [189]. The mode of action of QACs is through electrostatic interaction between the negatively charged bacterial membrane and the positively charged QAC. Following the electrostatic interaction, the structural proteins and enzymes of the bacterial membrane are denatured owing to the integration of the hydrophobic tail of the QAC into the hydrophobic membrane. Nanoscale materials immobilized with *N*-alkylated polyethyleneimine (PEI) have bactericidal activity against Gram-positive and Gram-negative bacteria (water and airborne), and fungi, including antibiotic-resistant strains, through cell membrane rupture [186]. Substitution of PEIs with various groups also results in activity against *C. albicans.* In a report by Wan and Yeow [190], amine-functionalized TiO_2_ and AuNPs were shown to have excellent antibacterial properties without external excitation. In another study, quaternary ammonium compounds functionalized with SiNPs were reported to have improved bactericidal activity against *E. coli* (96.6%), *S. aureus* (98.5%), and *Deinococcus geothermalis* (99.6%) in comparison with pristine SiNPs [191]. Likewise, QAC-PEI-based NPs completely inhibited the growth of *S. aureus* at 80 g/mL, and *E. coli* at 320g/mL [192].

#### 2.2.2. Triclosan and Polysiloxanes

Triclosan exhibits antimicrobial activity against *Corynebacterium* [193], and is one of the most commonly used antimicrobial agents. Polysiloxanes are linear polymers of silicon oxide. Siloxane copolymers substituted with quaternary ammonium groups are effective against *E. coli* and *S. aureus* [194].

#### 2.2.3. Chitosan

Chitosan NPs exhibit a wide spectrum of activity against bacteria, viruses, and fungi. The characteristic features of chitosan (biocompatibility, non-toxicity, low-immunogenicity, antibacterial activity, and its ability to enhance absorption) increase its applicability in various fields [186,195]. The antibacterial activity of chitosan NPs depends on various factors including the pH and solvent [186]. Chitosan NPs exhibit good antifungal activity against *C. albicans* and *Fusarium solani.* However, *A. niger* is relatively resistant to chitosan NPs. The zeta potential is believed to have an effect on the negatively charged microbial surface, and contributes to the antifungal effect of chitosan [196].

### 2.3. Antiviral Properties of NPs

Viruses are one of the leading causes of disease and death globally. They can infect all life forms from prokaryotes to eukaryotes. The emergence of new resistant viral strains has become a global challenge for physicians and scientists alike. Vaccination programs have eradicated many viral diseases such as smallpox and paralytic poliomyelitis. Nanotechnology offers intriguing opportunities to overcome the problems associated with antiviral drug resistance through the re-exploration of known antimicrobial compounds by manipulation of their size [197]. Metal NPs kill various viruses such as human immunodeficiency virus 1 (HIV-1) [198,199,200], the hepatitis B virus [201], the influenza virus [202,203,204], the *Tacaribe virus* [205], the monkeypox virus [206], the respiratory syncytial virus [207], and herpes simplex virus type 1 (HSV-1) [208,209]. Although the mechanism of action of NPs is not clearly understood, reports indicate that NPs prevent infection by blocking viral entry into cells by competing with cellular heparan sulfate, and interfering with the beginning of the viral replication cycle [197,199,200]. AgNP/chitosan composites have antiviral activity against the H1N1 influenza A virus; however, chitosan alone did not exhibit any antiviral activity [210]. The inhibitory effect of AgNPs was attributed to the inhibition of virus attachment to the cell surface, and might involve modification of the viral protein by denaturation of its disulfide-bonded domain [197]. AuNPs stabilized with PEG have antiviral activity against HIV-1 and inhibit virus fusion; however, the mechanism of action is unclear [211]. Research has demonstrated that AuNPs bind with gp120, inhibit viral entry, and prevent CD4 attachment [211]. A study by Broglie et al. (2015) demonstrated the antiviral activity of a Au/copper sulfide core/NP shell (Au/CuS/NPs) system against norovirus virus-like particles (VLPs) [212]. Au/CuS/NPs may work by inactivating VLPs by direct contact, then destroying the capsid. The authors of an earlier study reported that copper and copper alloys degrade the GII.4 human norovirus genome and destroy the capsid [213]. Hence, it is believed that the copper component of Au/CuS/NPs may play a significant role in its antiviral activity. Copper iodide (CuI) NPs exhibit excellent antiviral activity against feline calicivirus; this may be due to Cu(^+^) ROS generation followed by the oxidation of capsid proteins [214]. It has been reported that the nanocomposite comprising TiO_2_, poly-l-lysine (PL), and DNA/RNA (TiO_2_, PL, DNA/RNA) exhibits antiviral activity against influenza A virus (H3N2 strain), as demonstrated by antisense technology [215].

## 3. Biological Compatibility of Nanoparticles (NPs)

The biological compatibility, or biocompatibility, of nanomaterials is their ability to function within a host system such that the host responds in an appropriate way in specific situations. Advances in nanotechnology have made possible the use of engineered NPs in the treatment of diseases. However, prior to the use of NPs in biomedical applications, their biological compatibility must be established [216]. Direct contact with cells, tissues, the extracellular environment, and physiological systems can trigger a sequence of biological effects that can be either beneficial or destructive. The physicochemical properties of NPs determine their biological compatibility as well as their efficacy. Hence, the establishment of standard evaluation criteria for biological compatibility is required for the biomedical application of NPs. The biological compatibility or toxicity of NPs can be studied with respect to cytotoxicity, hemocompatibility, histocompatibility, and neurotoxicity. NPs come into direct contact with blood when they are used as vectors in gene delivery, drug delivery, or as biosensors. Hence, the evaluation of blood-NP compatibility is essential before they can be used. Several techniques including hemolysis, blood cell aggregation, and coagulation behavior studies are available for the evaluation of blood-NP compatibility [217]. Blood-NP compatibility depends on several factors such as size, structure, and the surface properties of the NPs [217]. The increased and/or widespread use of NPs in biomedicine raises concerns about their access to different tissues and organs. NPs play a significant role in targeted drug delivery and diagnostics, and they have been explored intensively. However, histocompatibility needs to be evaluated prior to their application. Several NPs including dendrimers, AuNPs, carbon nanotubes, and superparamagnetic Fe_3_O_4_ (SPIONs) have been evaluated for histocompatibility. Both in vitro and in vivo toxicity studies have revealed that SPIONs are biocompatible and do not exhibit any severe toxic effects [218]. Silica-based NPs, which are known to be highly compatible, enter the cell without affecting cell survivability [217].

AgNPs induce cell toxicity by releasing Ag^+^ that cause cell shrinkage, affect membrane integrity, and induce apoptosis [219,220,221]. Research clearly indicates that AgNPs release free radicals, which induce cellular and DNA damage [222]. In addition, AgNPs show immunotoxicity in rats [223]. However, AgNPs are biocompatible and non-toxic under certain experimental conditions, and they are suitable for biological applications [224]. An earlier study revealed that cells treated with AgNPs in the presence of hydrogen peroxide exhibited no DNA damage [225]. According to earlier studies, based on inhalation or oral exposure, there was no severe toxicity from AgNPs in rat organs in vivo [223,226,227,228]. This was attributed to low absorption of the AgNPs from the lung and gastrointestinal tract. Munger et al. [229] reported that in vivo oral exposure to a commercial AgNP solution resulted in no clinical changes in human metabolism, hematology, urine, and imaging morphology. In vitro studies have indicated that the cell toxicity of AgNPs arises at concentrations of 5–50 μg/mL [230]. AgNP-biopolymer composites showed good antibacterial activity with no toxicity against three eukaryotic cell lines, namely, mouse fibroblasts (NIH-3T3), human osteosarcoma cells (MG63), and human hepatocarcinoma cells (HepG2) [231]. A 3-(4,5-dimethylthiazol-2-yl)-2,5-diphenyltetrazolium bromide (MTT) assay and a cell adhesion test confirmed that Ag/chitosan nanocomposites are compatible with L-929 normal cells [232]. AuNPs are less harmful to RAW264.7 cells then AgNPs [233]. Negligible cytotoxicity was observed in the cell lines when treated with Fe_3_O_4_-AuNPs [234]. TiO_2_ NPs are reported to be non-toxic at low doses (5 mg/kg body weight) and efforts have been made to increase their cytocompatibility [235,236]. More recently, a review by Shi et al. [237] on the toxicity of mesoporous silica NPs emphasized their biocompatibility. Various studies on the immune compatibility of NPs have been discussed in detail elsewhere [235]. More recently, 4T1 cells (a murine breast cancer cell line) exposed to below 125 µg/mL of manganese ferrite (MnFe_2_O_4_) NPs showed biocompatibility [238]. According to Tomitaka et al. [239], various ferrite NPs (Fe_3_O_4_, ZnFe_3_O_4_, and NiFe_3_O_4_) above 100 μg/mL show toxicity against HeLa cell lines. Similarly, TiO_2_ NPs at lower concentrations (100 μg/mL) are considered harmless to humans based on in vivo studies [240]. CaFe_2_O_4_ NPs were shown to exhibit high toxicity when used at concentrations above 250 μg/mL [241].

The severity of NP cytotoxicity depends on the route of administration and the site of accumulation. Polymeric NPs, which are surface modifiable and are capable of sustained drug release, are considered biocompatible and are used in the treatment of various pulmonary diseases such as asthma, tuberculosis, and lung cancer [242,243]. Several reports have demonstrated the neurotoxic potential of NPs. However, the mechanism and the pathways by which NPs exert neurotoxicity remain largely unclear. The high degree of biological compatibility of NPs indicates that they interact with the system without causing any unacceptable toxic, carcinogenic, immunogenic, or thrombogenic reactions. The biological compatibility of NPs depends on many factors including their size, structure, and formulation [225,244]. Some of the most important factors that influence the biocompatibility of NPs are as follows. First, application: NPs used in a particular application may be toxic in specific tissue types. However, they may not produce the same effect in different applications/tissue types. Second, the exposure half-life of NPs, i.e., the intrinsic characteristics of NPs, does not exclusively determine their biocompatibility. Third, biological compatibility is a relative factor and depends on the risk–benefit ratio. Fourth, the lack of adequate data: at present, the understanding of the biological compatibility of NPs is limited owing to a lack of information [244]. Hence, more research is necessary to evaluate the biological compatibility of NPs in a tissue- and application-specific manner.

## 4. Biodegradability and Encapsulation of Nanoparticles

The internal digestion and subsequent clearance of NPs from the body is commonly referred to as biodegradability. Biodegradable NPs (BNPs) are more appropriate for biomedical applications than non-degradable NPs. The clearance of NPs from the body after they have fulfilled their function is highly significant. Non-biodegradable NPs may have toxic side effects caused by their accumulation in mononuclear phagocytic cells such as those found in the liver and spleen [244]. Advances in the development of BNPs have revolutionized medicine. Polymer-based BNPs are submicron-sized colloidal particles; they can harbor a therapeutic agent embedded/encapsulated within their polymeric matrix or adsorbed/conjugated onto their surface [245]. The characteristic features of BNPs, such as their bioavailability, their suitability for the controlled release of drugs, genes, and other bioactive agents, their high encapsulation capabilities, and their low toxicity, have increased their applicability to the site-specific delivery of vaccines, drugs, and many other bioactive molecules. Proteins, polysaccharides, and synthetic biodegradable polymers are the source materials for the synthesis of BNPs. The selection of basic polymers for the synthesis of BNPs depends on the design and end application of the NPs. Several other factors are also important, including the degree of biodegradability and biological compatibility, the surface characteristics and functionality, the size of the desired NPs, and the properties of the encapsulated drugs [246]. Several methods are available for the synthesis of BNPs, including the dispersion of preformed polymers, ionic gelation, and the polymerization of monomers.

Several different biodegradable polymer matrices have been extensively used in the preparation of BNPs; these include chitosans, poly-alkyl-cyanoacrylates (PAC), gelatins, poly-d,l-lactide-co-glycolides (PLGA), polylactic acids (PLA), and poly-ε-caprolactones (PCL). PLGA undergoes hydrolysis in the body to produce metabolically biodegradable products, namely lactic and glycolic acids. Several different methods, such as solvent evaporation, emulsification-diffusion, and nanoprecipitation, are available for the preparation of PLGA NPs [247]. PLGA NPs have been used in the development of nanovaccines, gene delivery systems, and protein- and peptide-based nanomedicines [247,248]. PLA is considered a biocompatible and biodegradable polymer, and it is broken down in the body to lactic acid. PLA NPs can be prepared by solvent displacement, solvent evaporation or diffusion, and salting-out. PCL undergoes hydrolysis under normal physiological conditions with minimal or no toxicity. Hence, PCL is a potential candidate polymer for the preparation of BNPs, with applications in long-term implantable devices and drug delivery [246]. Various techniques such as solvent displacement/evaporation and nanoprecipitation have been used to prepare PLC NPs [246]. Gelatin is a polyampholyte and is extensively used in medical and food products. Gelatin NPs may be prepared by emulsion or desolvation/coacervation methods. Gelatin NPs can be used for drug delivery and the controlled release of drugs. Moreover, these NPs are nontoxic, biodegradable, and bioactive. Upon biodegradation, PACs produce several toxic compounds that have the potential to damage or stimulate the central nervous system. Hence, the use of PAC polymers in humans is not authorized [246].

Macrophages/phagocytic cells are a significant part of the mononuclear phagocytic system, and are involved in the removal of foreign particles including NPs (which may be considered foreign particles) from the circulation. Hence, the surface modification of NPs is necessary to allow them to evade the immune system of the body and succeed in vivo [249]. Surface-modified NPs (modified with biomolecules) remain in the vascular system for a prolonged period. Hence, surface-modified NPs may reach their target site safely and rapidly in comparison with non-modified NPs [246]. Several different polymers are available for the surface modification of NPs, including PEG, polysorbate 80, polysorbate 20, dextran, and tocopheryl polyethylene glycol 1000 succinate. However, PEGylation is the most common technique. PEGylation is the process of grafting or adsorbing PEG onto the surface of NPs. This increases the blood circulation half-life of the NPs. An earlier study by Leroux et al. [250] demonstrated that PEGylated PLGA NPs interact less with mononuclear phagocytes and remain in the systemic circulation longer. A study by Tobio et al. [251] revealed the decreased conversion of PEG-PLA NPs into lactic acid compared with PLA NPs. Tocopheryl polyethylene glycol 1000 succinate-modified NPs exhibit increased adhesion towards tumor cell surfaces [246]. A nano-suspension of paclitaxel (an anticancer agent) bound to biologically compatible proteins such as albumin (the system is commercially known as Abraxane) has been approved for the treatment of breast cancer [252].

## 5. Nanoparticles and Delivery Systems

One of the major obstacles associated with the treatment of many diseases is the effective delivery of a therapeutic compound to the target site. Conventional approaches to drug delivery have several drawbacks and limitations such as poor biodistribution, limited effectiveness, and a lack of selectivity [253,254]. Controlled drug delivery systems may overcome the drawbacks and limitations of conventional approaches. Such systems transport the drug/molecule to the site of action, protect the drug from rapid clearance/degradation from the body, and reduce undesirable side effects. The site-specific delivery of a drug is achieved through the attachment of the therapeutic drug to the NPs, which act as carriers, known as nanocarriers. Nanocarriers are readily absorbed by cells owing to optimized physicochemical and biological properties; hence, they can be successfully used to deliver therapeutic/bioactive compounds [252,254]. Many different nanocarriers are available for the development of drug delivery systems, including liposomes, dendrimers, polymers, carbon materials, solid lipid NPs, silicon, and magnetic NPs. Polysorbate-coated NPs attached to doxorubicin, an anti-cancer drug, are capable of crossing the intact blood-brain barrier (BBB), and are used in the treatment of brain cancer [255]. Moreover, a multifunctional system, known as Probes Encapsulated by Biologically Localized Embedding (PEBBLE), in which a variety of unique agents/molecules are encapsulated on the surface of NPs to perform multiple functions, has been designed by Summer and Kopelman [256]. One molecule guides the PEBBLE to a tumor, a second molecule helps visualize the target (by MRI), while a third molecule delivers a drug to the nearby cancer cells [256].

NPs can also be used as vectors in gene delivery or as biosensors. It is well known that most viral genomes undergo point mutation and recombination, which may lead to the emergence of new resistant virus strains. The treatment of such diseases is difficult because available drugs become ineffective. In such a situation, antisense technology may allow the selection of conservative regions in virus genomes. Peptide analogues of nucleic acids (PNA), which form complementary base pairs with RNA, are one of the most suitable candidates for inhibiting gene expression in vivo [215]. The successful delivery of PNA into the target site inhibits the expression of a target gene. Amirkhanov et al. [215] prepared a nanocomposite comprising TiO_2_, PL, and DNA/RNA using an electrostatic fixation method. These nanocomposites are capable of penetrating eukaryotic cells and effectively delivering PNA. Such nanocomposites exhibit antiviral activity against influenza A virus (H3N2 strain); the results indicate that TiO_2_ acts as a carrier and plays a significant role in the delivery of PNA into the cells. Several different polymer-based NPs have been devised for the delivery of RNA; they include PLGA- and PLA-based NPs for in vitro RNA interference (RNAi) delivery [252,257]. Chitosan-based NPs with encapsulated quantum dots (QD) have been successfully used to deliver small interfering RNA (siRNA), targeting human epidermal growth factor receptor-2 (HER2/neu) [258]. Moreover, chitosan/QD NPs surface-labeled with the HER2 antibody have been used in the targeted delivery of HER2 siRNA to SKBR3 breast cancer cells, which overexpress HER2 [252]. NPs labeled with fluorescent markers, such as Cy-5, help visualize the uptake and accumulation of nanotubes. Moreover, smart superparamagnetic Fe_3_O_4_ particle conjugates may be used to target and locate brain tumors earlier and more accurately than is possible using current techniques [252,259].

## 6. Limitations

NPs can be synthesized by several different methods and do not exceed 100 nm in size. The size, chemical composition, and shape of these NPs depend on the method of synthesis. They may be organic or inorganic, and may comprise metal, polymer, ceramic, latex, or carbon-based particles [174]. However, the major challenge commonly encountered is to control the size and shape of monodispersed NPs with higher stability during synthesis. Interestingly, the interaction between NPs and living cells/tissues is affected by several factors, including their size, shape, and composition [217]. The ability of NPs to permeate is essential if they are to be useful as antimicrobial or drug-delivery agents [31]. The majority of the NPs synthesized to date have the capacity to permeate membrane cells and spread to different regions of the body, such as blood vessels, nerve cells, and the lymphatic vascular system. However, NPs have potential health threats owing to selective accumulation in different cells, tissues, and certain cellular structures [174,217].

Several groups have studied the effect of NPs on biological systems and have reported that exposure to NPs with a diameter less than 100 nm may pose known and unknown risks. Moreover, risks associated with NPs vary with the type of NP [260]. NPs may escape the immune defense mechanism of the body owing to their small size and may cause inflammatory and/or toxic responses [217]. Experimental studies have revealed the relationship between pulmonary inflammation and toxic responses to ultrafine particles. The high surface area of these fine particles allows them to interact with cellular structures and is believed to increase oxidative stress. The ROS generated by NPs are major contributors to inflammation and toxicity, inducing oxidative stress, apoptosis, and the activation of signaling pathways, which may lead to the development of many conditions, such as pulmonary diseases. Moreover, exposure to NPs cause lung inflammation [261,262]. The translocation of ultrafine particles to extrapulmonary sites such as the liver, heart, and brain, and even to the systemic circulation, has been observed recently in both human and animal studies [261,262]. The effect of NPs on biological systems is not completely known. Hence, the harmful effects and limitations of NPs should be carefully investigated.

## 7. Conclusions

The evolution of drug-resistant microorganisms poses a great challenge for medical practitioners, and new drugs are urgently sought to treat many diseases. Microorganisms develop drug resistance by various mechanisms. Innovative advances in nanomedicine offer the possibility of NP-based bioimaging and early detection systems, and the diagnosis and treatment of diseases caused by drug-resistant microorganisms. The development of technology and techniques for the synthesis of NPs/nanocomposites has also revolutionized the field of biomedicine. Several such NPs have been developed by different research groups, and their antimicrobial activity has been tested on different microorganisms. The synthesis of NPs by biological methods reduces the environmental concerns associated with chemical synthesis. Several research groups have developed biological techniques for the synthesis of NPs that are environmentally friendly. The mode of action of NPs varies with the type of NP, their size, and composition. NPs have found applications in several other fields of biomedicine such as bioimaging, drug delivery, gene delivery, and cancer therapy. Though NPs have significant applications in biomedicine, the limitations, and/or health risks associated with these nano-sized particles cannot be ignored. Concrete guidelines need to be devised by the experts from industries, governments, and the scientific community to reduce the toxicity and other unpredictable effects of NPs on human and animal health. Although NPs pose some drawbacks, they provide hope for the development of effective antimicrobial agents for the future.

## 8. Future Prospects

NPs/nanocomposites have provided some hope for the treatment of many diseases that are difficult to manage/treat by currently available techniques. However, certain issues need to be addressed before NPs/nanocomposites can be used safely and effectively. Such issues include a more detailed understanding of the mechanism of action of NPs/nanocomposites, the development of eco-friendly methods for their synthesis, and the environmental and social implications of their use.

## Figures and Tables

**Table 1:** Mode of action of various nanoparticles/nanocomposites against pathogenic microbes.

Type of Nanoparticles	Mode of Action	Susceptible Microbes	References
Silver (Ag) nanoparticles	Interfere with the electron transport chain and transfer of energy through the membrane. Inhibit DNA replication and respiratory chain in bacteria and fungi.	Methicillin-resistant *Staphylococcus aureus*, *Staphylococcus epidermidis.* Vancomycin-resistant *Enterococcus faecium* and *Klebsiella pneumoniae*	[31,57,65]
Magnesium oxide (MgO) nanoparticles	Formation of reactive oxygen species (ROS), lipid peroxidation, electrostatic interaction, alkaline effect.	*S. aureus*, *E. coli*, *Bacillus megaterium*, *Bacillus subtilis*	[66,67]
Titanium dioxide (TiO_2_) nanoparticles	Formation of superoxide radicals, ROS, and site-specific DNA damage.	*E. coli*, *S. aureus*, and also against fungi	[28,31,68,69]
Zinc oxide (ZnO) nanoparticles	Hydrogen peroxide generated on the surface of ZnO penetrates the bacterial cells and effectively inhibits growth. Zn^2+^ ions released from the nanoparticles damage the cell membrane and interact with intracellular components.	*E. coli*, *Listeria monocytogenes*, *Salmonella*, and *S. aureus*	[70,71,72,73,74]
Gold (Au) nanoparticles	Generate holes in the cell wall. Bind to the DNA and inhibit the transcription process.	Methicillin-resistant *S. aureus*	[75,76,77,78]
Copper oxide (CuO) nanoparticles	Reduce bacteria at the cell wall. Disrupt the biochemical processes inside bacterial cells.	*B. subtilis*, *S. aureus*, and *E. coli*	[79,80,81,82]
Iron-containing nanoparticles	Through ROS-generated oxidative stress. ROS, superoxide radicals (O^2−^), singlet oxygen (^1^O_2_), hydroxyl radicals (OH^−^), and hydrogen peroxide (H_2_O_2_).	*S. aureus*, *S. epidermidis*, and *E. coli.*	[83]
Aluminum (Al) nanoparticles	Disrupt cell walls through ROS.	*E. coli*	[82,84]
Bismuth (Bi) nanoparticles	Alter the Krebs cycle, and amino acid and nucleotide metabolism.	Multiple-antibiotic resistant *Helicobacter pylori*	[85,86]
Carbon-based nanoparticles	Severe damage to the bacterial membrane, physical interaction, inhibition of energy metabolism, and impairment of the respiratory chain.	*E. coli*, *Salmonella enteric*, *E. faecium*, *Streptococcus* spp., *Shewanella oneidensis*, *Acinetobacter baumannii*, *Burkholderia cepacia*, *Yersinia pestis*, and *K. pneumonia*	[87,88,89,90,91]

## Abbreviations

The following abbreviations are used in this manuscript:

Ag_2_O	Silver oxide
AgNPs	Silver nanoparticles
Al_2_O_3_	Alumina oxide
AuNPs	Gold nanoparticles
BINPs	Bismuth nanoparticles
BNPs	Biodegradable nanoparticles
CaO	Calcium oxide
CuO	Copper oxide
CuFe_2_O_4_	Copper ferrite
HIV-1	Human immunodeficiency virus 1
HSV-1	Herpes simplex virus type 1
HRTEM	High-resolution transmission electron microscopy
LRTEM	Low-resolution transmission electron microscopy
MBC	minimum bactericidal concentration
MIC	Minimum inhibitory concentration
MgO	Magnesium oxide
MnFe_2_O_4_	Manganese ferrite
MRSA	Methicillin-resistant *Staphylococcus aureus*
NPs	Nanoparticles
PAC	Poly-alkyl-cyanoacrylates
PCL	Poly-ε-caprolactone
PEG	Polyethylene glycol
PEI	*N*-alkylated polyethyleneimine
PLGA	Poly-d-l-lactide-co-glycolide
PLA	Polylactic acid
PL	Poly-l-lysine
PNA	Peptide analogues of nucleic acids
QAC	Quaternary ammonium compounds
RNAi	RNA interference
ROS	Reactive oxygen species
siRNA	Small interfering RNA
TiO_2_	Titanium dioxide
TiO_2_NPs	Titanium dioxide nanoparticles
TEM	Transmission electron microscopy
TPGS	Tocopheryl polyethylene glycol 1000 succinate
ZnO	Zinc oxide
ZnFe_2_O_4_	Zinc ferrite
